# School Commissioners State Association to Cooperate with State Board of Health

**Published:** 1904-06

**Authors:** M. L. Duggan

**Affiliations:** Secretary Convention; Dublin, Ga.


### EDITORIAL NOTES AND COMMENTS.

The Business office of THE JOURNAL-RECORD is No. 1007-1008 Century Building.

The Editorial office is 318-319-320 The Prudential.

Address all Business communications to Dr. M. B. Hutchins, Mgr.

Make remittances payable to THE ATLANTA JOURNAL-RECORD OF MEDICINE.

On matters pertaining to the Editorial and Original Communications address Dr. Bernard Wolff, Atlanta.

Reprints of original articles will be furnished at cost price. Requests for the same should always be made *on the manuscript*.

We will present, post-paid, on request, to each contributor of an original article, twenty (20) marked copies of THE JOURNAL-RECORD containing such article.

### SCHOOL COMMISSIONERS STATE ASSOCIATION TO COOPERATE WITH STATE BOARD OF HEALTH.

DUBLIN, GA., May 6.—(Special.)

At the Wednesday night's session of the county school commissioners in this city, Dr. Charles Hicks, Vice-President of the State Board of Health, upon invitation, delivered a short address upon the importance of cooperation between the school officials and the State health authorities. After the address a motion was unanimously passed indorsing the suggestions made, and pledging the assistance of the commissioners in the effort to put the schools of the State on the best possible hygienic basis. Dr. Hicks' address in part was as follows:

"A 'Greater Georgia' means advancement along all lines. The highest evidence of progressive civilization is the demand for information. In obedience to the popular demand, the General Assembly of our commonwealth within the last year has created another State department—the Department of Health; its functions to be performed by the State Board of Health and the county and municipal boards of health throughout the State. The construction of the department of health, patterned largely after the construction of the department of education, the functions of which are performed by a State school commissioner and the county and municipal boards of education. The department of health embraces in its organization other than members of the medical profession, the mayors of cities and towns and the ordinaries and chairmen of commissioners of roads and revenue, in order that the expenses of this department may be properly provided for when fully organized in the various counties throughout the State.

### POINTS TO RESULTS.

"That you may note the efficient service under public health laws, I point you to results. All of you are doubtless familiar with the salutary effects of public health laws as applied at Ithaca, New York State. As you know, it is the school city of that State. Ithaca is to New York what Athens is to Georgia. Two or three years ago an epidemic of typhoid fever broke out among the students domiciled at Ithaca attending the various institutions of learning. Misunderstandings arose between the city authorities, the various faculties and the local boards of health; the governor of the State had representatives of the State Board of Health go to Ithaca, look into the conditions relative to the presence of typhoid fever, and to report without delay its eradication. The source of the disease was located, the cause removed and the malady eradicated without commotion.

"You recall that just after the Spanish-American war the American troops, led by sanitary experts, did as much and greater service for humanity in clearing the cities of Cuba of infectious diseases, than when led by military captains they drove the Spaniards from the island. Recall, if you please, the extraordinarily large expenditures by the municipalities of your seaport cities and the calamities that followed fast on epidemics, until in more recent years the general government created the marine hospital service to bar the importation of infection, since which time yellow fever nor any other infection has brought panic to any of our ports or shores. While the maritime laws protect our coast from infection and epidemics in a large measure, yet no municipality in Georgia to-day, however well provided for by appropriations from their treasuries, nor however well guarded by apprehensions and health officers, can remain immune against infection from transportation lines, the smaller towns and rural districts, most of which are without the protection of sanitary regulations. The States about us have sanitary regulations with working organizations which are effective in preventing persons with infectious and contagious diseases, or those who have been exposed to contagion, landing on or passing through their domain, but up to a year ago we have had no State law to prevent influx from localities in those States where contagious disease is declared to exist.

"You have but to recall five years ago when localities in the State of Alabama were infected and one of the transportation lines from that State poured smallpox into the smaller towns and the rural districts in Southern Georgia. This infection has remained here ever since, a smouldering volcano. The railroads were helpless, our State officials were powerless. And now, without the support of such organizations as yours, progress will be retarded and calamity possibly added to misfortune. The prophylaxis vaccination, although known and successfully demonstrated for one hundred years, is not generally believed in, to our shame.

SMALLPOX WORST EPIDEMIC.

"There is no disease which can so thoroughly break up business in any city or community, in the event of an epidemic, as smallpox, since this disease is as sensational as it is dangerous and contagious.

"There is no other means given under heaven among men whereby we can be saved from the ravages of smallpox, except by successful vaccination, quarantine and fumigation to be rigidly applied when the disease finds a lodgement.

"It is not necessary to consume time in arguing, before such an intelligent and enlightened body as this is, the needs for a State Department of Health. It is my purpose here this evening to ask the State School Commissioner, this association and your teachers throughout the State to cooperate with the State Board of Health in the application and securement for Georgia of a perfect system of hygienic and sanitary laws.

"In return we offer you the results, which will redound not only in the general welfare of the State, but in harmonious cooperative work these two departments will add greatly to the improvement in environment of every school building in the State, and add to the material progress and advancement of every school in Georgia."—*Report Atlanta Constitution.*

Resolutions passed by county school commissioners assembled in annual convention at Dublin, Ga., May, 1904, after a short address by Dr. Chas. Hicks, member State Board of Health:

Recognizing the relationship existing between the State department of health and the State department of education, and appreciating the great good accomplished, and to be accomplished by the Georgia State Board of Health; be it therefore

*Resolved, First,* That this association in convention assembled, tender the State Board of Health its hearty cooperation in the furtherance of the work begun in the suppression of contagious and infectious diseases in our State.

*Second,* That the State School Commissioner, and the various county school commissioners, direct the teachers of the different schools of the State to conform to, and lend their aid to the various local boards of health, in carrying into effect the public health laws.

*Third,* That the members of this association not only aid in the application of the present laws, but also aid in the procurement of a perfect system of hygienic and sanitary laws for Georgia.

Unanimously adopted,

(Signed) M. L. DUGGAN, Secretary Convention.

The following resolution was unanimously adopted by the Medical Association ot Georgia, at its fifty-fifth annual meeting, held in the city of Macon, May 20th–22d, 1904, after the reading of the report of the special committee on the State department of health.

WHEREAS, The early organization of the county boards of health in counties not having them in this State being necessary for systematic and thorough working of the State Board of Health; and it being essential that the public be educated to the necessity for its full sympathy and support of the profession in the State being essential for a speedy envolvement of an efficient system of public health laws; be it therefore

*Resolved*, That this Association pledge its support in the furtherance of legislation looking to the good of the State in this regard, and do recommend that a conference of the State Board of Health and of all county and municipal boards of health be organized, and that this organization meet annually at the same time and place that the Medical Association of Georgia holds its annual meeting, said organization's hours of meeting not to conflict with the hours of the meeting of the Medical Association.

